# Health-Related Complications of Acromegaly—Risk of Malignant Neoplasms

**DOI:** 10.3389/fendo.2019.00268

**Published:** 2019-04-30

**Authors:** Marek Ruchala, Kosma Wolinski

**Affiliations:** Department of Endocrinology, Metabolism and Internal Medicine, Poznan University of Medical Sciences, Poznań, Poland

**Keywords:** acromegaly, cancer, thyroid cancer, colorectal cancer, breast cancer

## Abstract

The issue of increased risk of benign and malignant neoplasms in patients with acromegaly remains the topic of debate from many years and was addressed by numerous studies. Many of them have shown increase in the cancer incidence. Among particular types of malignancies, thyroid, colorectal, and breast cancer are most commonly indicated as associated with acromegaly. Single reports denoted increase in prevalence of neoplasms of other organs such as kidney, bone or central nervous system. Cardiovascular and respiratory tract disorders were traditionally consider as main causes of mortality in acromegalic patients, accounting for about 60 and 25% of deaths, respectively. However, according to a number of studies published over the current decade cancer became the most important cause of deaths. Aim of the current article was to review the literature concerning the risk of malignant neoplasms in acromegaly and its clinical implications.

## Introduction

The issue of increased risk of benign and malignant neoplasms in patients with acromegaly remains the topic of debate from many years and was addressed by numerous studies. The most commonly discussed mechanism of such phenomenon is increased level of growth hormone (GH) and subsequently insulin-like growth factor 1 (IGF-1) (1). Both normal epithelial cells and colorectal cancer cells express IGF-1 receptor (IGF-R) and can be influenced by high levels of IGF-1 (1, 2). Experimental studies proved that IGF-1 directly stimulates proliferation of animal thyroid cells and potentiates proliferative effect mediated by TSH (1, 3). Also the positive correlation between the level of circulating IGF-1 and the risk of colorectal, breast or thyroid cancer had been demonstrated in general population (4, 5). Also some genetic alterations can be consider, especially in patients with genetic syndromes such as MEN 1 (6). Despite the pathophysiological mechanisms of carcinogenesis in acromegaly seem to be well-described, the clinical importance of this findings remains the issue of debate. Our aim was to perform the narrative review of the literature concerning the risk of malignant neoplasms in acromegaly and its clinical implications.

## Overall Cancer Incidence

The overall risk of malignant neoplasms in acromegalic patients was analyzed by numerous studies starting from the one published by Mustacchi et al. (7) in the fifties. No increase in the risk of malignant neoplasms was found, probably due to the fact that mortality was dominated by cardiovascular disorders at that time (1, 7). Next papers with similar construction has been published after about 30 years. Studies from the turn of eighties and nineties brought unequivocal results. Nabarro (8) found increase in the cancer risk in women (mainly due to increased prevalence of breast cancer) and no increase in the risk in men. Barzilay et al. (9)—increase of borderline significance. Ron et al. (10) investigated group consisted only of male patients; the study revealed elevated prevalence of malignancies (standardized incidence ratio [SIR] 1.6 with 95% confidence interval [95% CI] 1.3–1.9). Taking into account further studies, only three of them did not show significant increase in the risk of malignancies (11–13), whereas most indicated significantly elevated prevalence (14–20). The highest effect sizes were achieved in case-control studies performed by Popovic et al. (17) and Wolinski et al. (20)—odds ratios 3.2 and 3.3, respectively, in comparison to the control groups. Meta-analysis performed by Dal et al. (15) confirmed increase in overall cancer incidence with pooled SIR equal to 1.5 (95% CI 1.2–1.8).

## Thyroid Neoplasms

Risk of benign and malignant thyroid lesions was the topic of numerous studies. Wolinski et al. meta-analyzed available studies in 2014 (21). As it was described by the authors, however the amount of articles concerning the issue was high (22 initially selected by literature search), only five studies comparing the prevalence of thyroid cancer and three studies comparing the prevalence of benign thyroid lesions with sex- and age-matched control groups were available. Despite the limited amount of data, significantly increased risk of benign as well as malignant neoplasms was proved. Odds ratio (OR) for thyroid cancer was 7.87, for benign lesions−3.61. It was also checked if the risk of thyroid cancer is increased proportionally to the increased prevalence of thyroid lesions or the malignancy rate is higher in acromegalic patients. Results were unequivocal as the relative risk (RR) of malignancy for lesions in patients with acromegaly in comparison to the lesions in patients from control group was relatively high−3.1. However, the result was not statistically significant (95% confidence interval 0.5–20.1). The pooled prevalence of thyroid cancer and thyroid lesions meta-analyzed using random effect model was 4.3 and 59.2%, respectively. Furthermore, authors noticed higher prevalence of nodular goiter and thyroid cancer in newer studies. E.g., the pooled prevalence of thyroid cancer was about 3% in the studies published before 2008 and about 6% taking into account studies published since that year.

Update to the meta-analysis mentioned above was published in 2017 by the same authors (22); analysis was enriched by authors' own results and one additional case-control study (23). Main conclusions remained the same, however the effect size values slightly decreased—OR for thyroid nodules dropped from 3.6 to 3.1, for thyroid cancer—from 7.9 to 4.5, respectively. Also the issue of increased rate of malignancy remained unclear (RR was 2.3 with 95% CI 0.9–6.1 with *p* = 0.08).

Next meta-analysis concerning the issue was published by Dal et al. (15). Methodology of the study was described very briefly, flowcharts of literature search as well as precise inclusion and exclusion criteria were not given, so some aspects remains not fully elucidated. The aim of the study was to meta-analyze SIRs taken from available studies. That suggest inclusion of studies comparing prevalence in studied groups with expected prevalence in local populations. However, e.g., case-control study conducted by Wolinski et al. (20) was included whereas some studies with very similar methodology (17) were not. The selection criteria seem to be less strict than in two meta-analyses mentioned above. E.g., in included study performed by Wolinski et al. (20) all acromegalic patients as well as all patients from control group underwent thyroid ultrasonography (US) and subsequently fine-needle biopsy if indicated. In another included study performed by Orme et al. demographic data of acromegalic patients from 15 tertiary centers were used to search the national cancer registries (11). Such differences in methodology gave large differences in the results of included studies—in the first thyroid cancer was diagnosed in 7.0% of patients, in the latter in one patient in the group of 1239 subjects. Despite methodological differences, meta-analysis performed by Dal et al. (15) confirmed the increased prevalence of thyroid cancer in patients with acromegaly (pooled SIR equal to 9.2 with 95% CI 4.2–19.9).

## Colorectal Cancer and Polyps

Risk of colorectal neoplasms in patients with acromegaly was meta-analyzed for the first time by Rokkas et al. (24). The selection criteria were strict and well defined; finally nine case-control colonoscopic studies were included. However, only three of them compared the risk of colon cancer in acromegalic patients with control groups, eight studies included data on the risk of adenomatous polyps and seven—of hyperplastic polyps. According to the results of the meta-analysis all mentioned lesions were significantly more common in acromegalic patients. OR for colon cancers was 4.4, for adenomas−2.5, for hyperplastic polyps−3.7 (random effect model).

The next meta-analysis concerning the prevalence of colorectal cancer was published in 2018 (15). The selection criteria were not such strict and in fact all studies comparing cancer incidence in comparison to control groups were included, encompassing colonoscopic studies (25), retrospective studies analyzing medical documentation of the patients (20) as well as studies based on cancer registries (10). Less strict criteria resulted in higher number of included studies (fourteen papers). The pooled SIR was 2.6 (with 95% CI 1.7–4.0) confirming increase prevalence of colorectal cancer. There where large differences in SIRs between studies—from 1.4 without statistical significance (11) to 18.2 (26). Authors noticed also the difference between single-center studies (SIR 7.3) and multicenter or population-based studies (SIR 2.0 and 2.2, respectively).

## Breast Cancer

The issue of coexistence of acromegaly and breast cancer appeared in the literature almost 60 years ago; in the year 1960 a case of Hungarian women who developed breast cancer 2 years after the diagnosis of acromegaly with further recurrence of the neoplastic disease 9 years after the radical mastectomy was described (27). Probably nowadays publication of such case-report would be difficult. But do we know more about the association of acromegaly and breast cancer? In fact only few case-control studies had been published. In the study performed by Popovic et al. (17) 220 acromegalic patients (137 females) and 249 subjects from control group (153 females) were included. Breast cancer was observed in four patients in the former and only one in the latter group; prevalence in the studied group was insignificantly increased. In the study performed by Wolinski et al. (20) breast cancer was present in 5.4% of acromegalic women and none of the patients in the control group, giving statistically significant result. There were also only few studies assessing the breast cancer risk in the groups of acromegalic patients and comparing it to the estimated risk in the general population. First such study was probably published by Nabarro (8)—reporting 11 cancers among 123 women vs. 2.6 expected, *p* < 0.01. In the multicenter study performed by Terzolo et al. (14) 1,512 acromegalic patients (888 women and 624 men) had been included. Breast cancer was diagnosed in 22 patients (against 16.8 expected) giving insignificantly increased risk. In the study performed by Petroff et al. (12) the methodology was more sophisticated; the study was based on the German Acromegaly Registry encompassing 57 medical centers, however, for the purpose of the analysis seven largest centers were asked to contact their patients; the phone interviews were main source of information. The prevalence of breast cancer was compared with data from national cancer registry; it was insignificantly increased (16 cases vs. 13.4 expected, *p* = 0.55). In the study performed by Dal et al. (15) this type of cancer was present in nine patients (among 529 patients, 261 women) against 3.6 expected—the difference was not significant. Finally, the risk of breast cancer was assessed in the meta-analytic part of the study performed by Dal et al. (15). The pooled SIR was 1.6 with 95% CI 1.1 to 2.3—so slightly but significantly increased. However, as the mentioned meta-analysis included numerous calculations (of overall cancer prevalence and risk of numerous particular cancers) methodology of the analysis concerning breast cancer is described very generally. In fact it is even not clear which studies were included and calculations are not reproducible.

## Other Cancers

There were only few studies reporting the increased prevalence of malignancies different than the three described above. Few studies reported increased risk of kidney and urinary tract malignancies. E.g., according to population-based cohort study performed by Baris et al. (18) SIR for kidney cancer was 3.2 with 95% CI 1.6–5.5. Also large Italian multicenter study performed by Terzolo et al. (14) showed significant increase in kidney cancer risk (SIR 2.9 with 95% CI 1.6–5.3). Baris et al. (18) reported also increased risk of bone and central nervous system malignancies as well as small intestine tumors in patients with MEN 1 syndrome.

## Summary and Conclusions

On the basis of the described studies the risk of malignant neoplasms seems to be increased in patients with acromegaly. The strongest evidence is available for thyroid and colon cancer. Higher incidence of these two cancers was confirmed by numerous studies and at least two meta-analyses performed by different authors. Relatively strong evidence is available for breast cancer, increased risk was described by few studies and confirmed by meta-analysis performed by Dal et al. (15). Risk of neuroendocrine neoplasms seems to be increased in patients with MEN1 syndrome (6, 18). The increased prevalence of other malignant neoplasms was reported in single or few studies and remains controversial.

The controversies on the issue and divergent results of particular studies have numerous causes. First of them is rarity of the disease (28, 29). Secondly—as it was underlined in the meta-analysis published by Wolinski et al. (21)—however one could have impression of abundance of available studies, most of them do not allow for reliable assessment of the cancer risk in acromegalic patients. Authors of the mentioned meta-analysis identified 22 studies assessing the prevalence of thyroid cancer. Half of them did not include control group (8, 26, 30–38), two included control groups not matched by sex and age (39, 40), one assessed only the occurrence of palpable goiter (41). All in all, only five case-controls studies (9, 17, 42–44) and three studies using data from hospital or national registers in comparison to local cancer registries were identified (10, 11, 18). That indicates the need for high quality case-control studies or large multicenter analyses comparing the results to local epidemiological data; descriptive studies giving just amount of particular cancers in investigated group can be interesting but in fact will not make a progress to the debate.

Another important aspect is the fact that—due to limited amount of high-quality studies—authors of reviews and meta-analyses are using studies published over a long period of time. For example the oldest study used the in recent meta-analysis written by Dal et al. (15) was paper published Mustacchi et al. (7); also numerous articles from eighties and nineties have been included. Studies published within such long time interval are in fact difficult to compare. Obviously, methods of the diagnostics and therapy of acromegaly and comorbidities have been changed radically. Due to the better treatment of the disease itself and its complications causes of death in acromegalic patients changed. Probably the most important improvement in last decades was introduction of somatostatin analogs (SSAs) and growth hormone antagonists (GHAs); lower mortality in Italian cohort treated with SSAs and GHAs in comparison with Bulgarian cohort—in which these drugs were poorly available—was described by Colao et al. (45). Bolfi et al. (46) meta-analyzed studies concerning standardized mortality rates (SMRs) in acromegalic patients. As in the studies published before 2008 SMR was 1.76 (95% CI 1.52–2.40), in those published after 2008 it was only insignificantly increased (1.35 with 95% CI 0.99–1.85). The drop in mortality and improve in life expectancy results in change in most common comorbidities and causes of death. Cardiovascular and respiratory tract disorders where traditionally consider as most common causes of mortality in acromegalic patients, accounting for about 60 and 25% of deaths, respectively (1). According to numerous studies published over the current decade cancer became the most common cause of mortality in this group (13, 47–50). This trend is understandable; probably in the past most patients died from other causes before cancers could develop and especially become clinically relevant; improvement in the management of acromegaly itself and comorbidities and—subsequently—extended life expectancy are resulting in the increase in prevalence and mortality from malignant neoplasms—similarly as in the general population. What is more, according to the data from numerous—also recent—publications, the delay in the diagnosis of acromegaly (from first symptoms to the final diagnosis) is usually about 8–10 years (8, 51). In consequence, even patients treated successfully shortly after the diagnosis had been exposed for increased levels of GH and IGF-1 for a long time; that can result in elevated risk of malignancies.

Another issue is the clinical relevance of the increased risk of malignancy. As cancer became recently the most common cause of mortality in patients with acromegaly, the importance seems to be obvious. What is more, taking into account recent publications, this trend will probably strengthen. There were some suggestions in the literature that especially small and asymptomatic thyroid cancers are overdiagnosed and have probably no impact on the survival (29, 52). It is well known that differentiated thyroid cancer is in general characterized by relatively slow growth, low mortality and very good prognosis (53, 54). However, in our opinion, it is difficult to assume that it is probably better not to diagnose concomitant thyroid cancer as the diagnosis could be the reason of poorer quality of live (29). Another issue are reasonable guidelines of diagnostics and therapy, adequate to the size of thyroid lesions/stage of thyroid cancer and the general patient's condition (55).

According to the current Endocrine Society guidelines (28) colonoscopy should be performed at diagnosis of acromegaly, thyroid US if there is palpable thyroid nodularity. In our opinion thyroid US and screening for breast neoplasia (US or mammography depending on the age) in women should be also performed at the baseline. Increased risk of these malignancies as well as low cost and invasiveness of screening test support the thesis.

To sum up, numerous studies proved increase prevalence of malignancies in patients with acromegaly; particularly elevated risk of thyroid, colon and breast cancer was indicated by many researches. Careful screening for malignancies should be important part of the management of acromegalic patients.

## Author Contributions

All authors listed have made a substantial, direct and intellectual contribution to the work, and approved it for publication.

### Conflict of Interest Statement

The authors declare that the research was conducted in the absence of any commercial or financial relationships that could be construed as a potential conflict of interest.
